# Activated Protein C Protects against Murine Contact Dermatitis by Suppressing Protease-Activated Receptor 2

**DOI:** 10.3390/ijms23010516

**Published:** 2022-01-03

**Authors:** Meilang Xue, Haiyan Lin, Ruilong Zhao, Callum Fryer, Lyn March, Christopher J. Jackson

**Affiliations:** 1 Sutton Arthritis Research Laboratory, Institute of Bone and Joint Research, Kolling Institute, Faculty of Medicine and Health, University of Sydney at Royal North Shore Hospital, St Leonards, NSW 2065, Australia; Haiyan.lin@sydney.edu.au (H.L.); rzha9073@uni.sydney.edu.au (R.Z.); callum.fryer1@gmail.com (C.F.); chris.Jackson@sydney.edu.au (C.J.J.); 2 The Australian Arthritis and Autoimmune Biobank Collaborative (A3BC), Institute of Bone and Joint Research, Kolling Institute, Faculty of Medicine and Health, University of Sydney at Royal North Shore Hospital, St Leonards, NSW 2065, Australia; lyn.march@sydney.edu.au

**Keywords:** activated protein C, atopic dermatitis, contact hypersensitivity, 1-fluoro-2,4-dinitrobenzene, protease activated receptor, cytokines, Th cells, mast cells, keratinocytes

## Abstract

Atopic dermatitis (AD) is a chronic inflammatory skin disease associated with excessive inflammation and defective skin barrier function. Activated protein C (APC) is a natural anticoagulant with anti-inflammatory and barrier protective functions. However, the effect of APC on AD and its engagement with protease activated receptor (PAR)1 and PAR2 are unknown. **Methods:** Contact hypersensitivity (CHS), a model for human AD, was induced in PAR1 knockout (KO), PAR2KO and matched wild type (WT) mice using 2,4-dinitrofluorobenzene (DNFB). Recombinant human APC was administered into these mice as preventative or therapeutic treatment. The effect of APC and PAR1KO or PARKO on CHS was assessed via measurement of ear thickness, skin histologic changes, inflammatory cytokine levels, Th cell phenotypes and keratinocyte function. **Results:** Compared to WT, PAR2KO but not PAR1KO mice displayed less severe CHS when assessed by ear thickness; PAR1KO CHS skin had less mast cells, lower levels of IFN-γ, IL-4, IL-17 and IL-22, and higher levels of IL-1β, IL-6 and TGF-β1, whereas PAR2KO CHS skin only contained lower levels of IL-22 and IgE. Both PAR1KO and PAR2KO spleen cells had less Th1/Th17/Th22/Treg cells. In normal skin, PAR1 was present at the stratum granulosum and spinosum, whereas PAR2 at the upper layers of the epidermis. In CHS, however, the expression of PAR1 and PAR2 were increased and spread to the whole epidermis. In vitro, compared to WT cells, PAR1KO keratinocytes grew much slower, had a lower survival rate and higher para permeability, while PAR2KO cells grew faster, were resistant to apoptosis and para permeability. APC inhibited CHS as a therapeutic but not as a preventative treatment only in WT and PAR1KO mice. APC therapy reduced skin inflammation, suppressed epidermal PAR2 expression, promoted keratinocyte growth, survival, and barrier function in both WT and PAR1KO cells, but not in PAR2KO cells. **Conclusions:** APC therapy can mitigate CHS. Although APC acts through both PAR1 and PAR2 to regulate Th and mast cells, suppression of clinical disease in mice is achieved mainly via inhibition of PAR2 alone. Thus, APC may confer broad therapeutic benefits as a disease-modifying treatment for AD.

## 1. Introduction

Atopic dermatitis (AD) is a chronic inflammatory skin disorder affecting up to 20% of children and 10% of adults worldwide [1]. AD is caused by repeated skin exposure to contact allergens [2], and characterized by excessive inflammation and defective epidermal barrier function [3]. The inflammatory response is distinguished by abnormal T cell activation, specifically Th1, Th2, Th17 and Th22 cells, depending on stage, severity, and disease subtype [4]. T cells then act on skin cells such as keratinocytes by cytotoxic effector mechanisms leading to tissue damage. Barrier disruption increases the invasion of allergens and causes more severe inflammation. Common current therapies for AD include glucocorticosteroids and non-steroid anti-inflammatory drugs; however, these therapies are often not fully effective and carry the risk of severe adverse effects, especially when used long-term or in children [5]. Consequently, there is a pressing need for the development of new and effective therapies for AD.

Activated protein C (APC) is a natural anticoagulant with a broad range of cytoprotective actions including inhibition of inflammation, stabilization of the endothelial and epithelial barrier and suppression of cell apoptosis [6]. Acute inflammation and barrier disruption are exacerbated in mice genetically predisposed to severe APC deficiency [7]. Recombinant APC shows therapeutic effects in various immunoinflammatory disease models [8], including allergic asthma [9,10] and diabetes [11]. These protective functions of APC are mainly mediated by cleavage of protease activated receptors (PARs) [12].

PARs are G-coupled transmembrane receptors. Among four members, PAR1 and PAR2 are expressed by most immune cells, epithelial and endothelial cells, and exert important roles in the regulation of innate and adaptive immunity [3,13,14]. In the skin, both PAR1 and PAR2 are abundantly present in the epidermis and dermis [15,16]. Although activation of PAR1 has been shown to promote the proliferation of human keratinocytes [17] and dermal fibroblasts [18], the functional consequence of PAR1 expression in the skin still remains elusive. In contrast, PAR2 plays multifaceted roles in epidermal homeostasis, barrier formation, immune responses [19,20,21,22,23], and the sensations of pain and itch [24]. Mice with epidermal overexpression of PAR2 develop AD-like symptoms [19], and inhibition of PAR2 activation suppresses inflammation and itch in both acute and chronic models of AD in mice [25].

The effects of APC and its engagement with PAR1 and PAR2 in AD are unknown. In this study, using PAR1 and PAR2 knockout (KO) mice and a 2,4-dinitrofluorobenzene (DNFB)-induced contact hypersensitivity (CHS) model for human AD [26], we demonstrated that APC therapy reduced the severity of CHS in matched wild type (WT) and to a less extent in PAR1KO mice, but not in PAR2KO mice. These data indicate that APC can mitigate CHS, and this function of APC occurs mainly by suppressing PAR2. 

## 2. Results

### 2.1. PAR2KO Mice Display Less Severe CHS and APC Mitigates CHS in WT and PAR1KO but Not PAR2KO Mice

**Figure 1:**
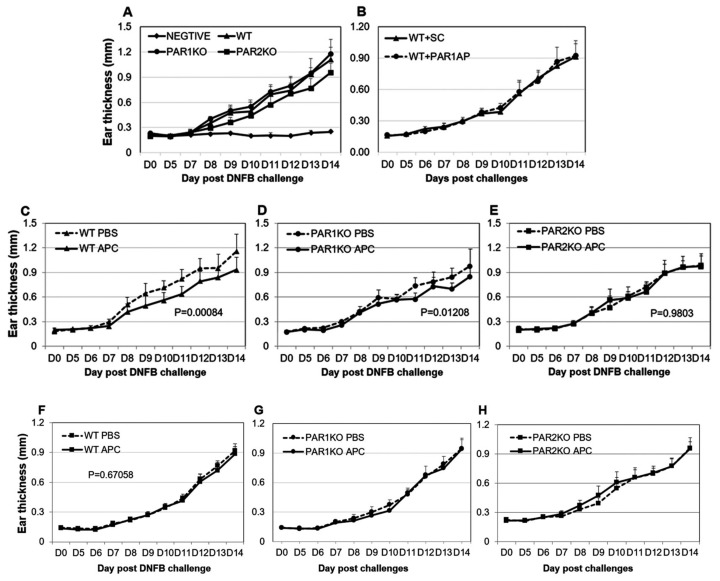
APC inhibits DNFB-induced ear thickness in WT and PAR1KO, but not in PAR2KO mice. CHS was induced via DNFB challenge on the flank skin on day zero and day one, then on the dorsum of both ears on day five to day 13 in mice. The ear thickness was measured from day zero to day 14. (**A**) The ear thickness of WT, PAR1KO and PAR2KO mice challenged with DNFB or solvent (NEGATIVE). (**B**–**E**) The ear thickness of mice challenged with DNFB and therapeutically treated with PAR1 activated peptide (WT + PAR1AP) or scramble control peptide (WT + SC) (10 mg/kg) in WT mice, APC (2 mg/kg) or PBS in WT (WT APC, WT PBS), PAR1KO (PAR1KO APC, PAR1KO PBS) or PAR2KO (PAR2KO APC, PAR2KO PBS) mice. (**F**–**H**) The ear thickness of WT, PAR1KO and PAR2KO mice challenged with DNFB and preventatively treated with APC (2 mg/kg) or PBS. Data shown on graph are means ± SD (*n* = 8 for all groups).

To investigate the effect of APC and PAR1/PAR2 on AD, a subchronic DNFB-induced CHS model was used to better reflect the chronic pathology of AD and to allow the therapeutic treatment of pre-established skin inflammation [26]. Clinical signs of CHS, including ear swelling, erythema and skin lesions rapidly developed after a four-day ear skin DNFB application in all mice. As expected, PAR2KO mice displayed an approximate 20% reduction (*p* = 0.0004) in ear thickness at day 14 when compared to WT mice, whereas no significant difference was found between PAR1KO and WT mice (Figure 1A). This suggests that PAR2 but not PAR1 is required for CHS development. This data was further confirmed by PAR1 agonist therapeutical administration, which showed that activation of PAR1 also did not affect DNFB-induced CHS (Figure 1B, data shown from 10 mg/kg).

APC significantly reduced ear swelling when used as a therapeutical treatment in WT (*p* = 0.001) (Figure 1C) and PAR1KO mice (*p* = 0.01) (Figure 1D), although the reduction in PAR1KO mice was less significant compared to WT mice. However, APC did not show this protective effect in PAR2KO mice (Figure E). These findings suggest that APC’s therapeutic effect on CHS does not depend on PAR1 and is likely via PAR2 suppression. In contrast with therapeutical treatment, APC did not display a significantly beneficial effect when applied as a preventative treatment as determined by ear thickness in mice (Figure 1F–H).

### 2.2. Effect of APC on Histopathological Features of CHS in Mice

**Figure 2:**
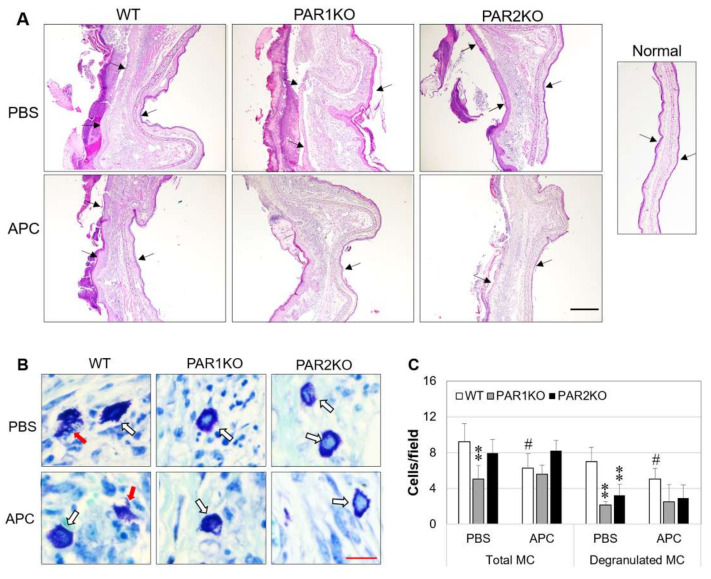
Histological features of DNFB-induced CHS in mice in response to APC therapy. Mice were euthanized at day 14 after DNFB challenge. Ear tissues were fixed, embedded, sectioned, and stained. (**A**) The representative images of ear skin tissues stained with hematoxylin and eosin. Arrows indicate epidermis. Scale bar: 500 µm. (**B**) The representative images of mast cells (MC) in the ear skin tissues of CHS, stained with toluidine blue. White arrows indicate non-degranulated MC and red arrows degranulated MC. Scale bar: 30 µm. (**C**) MC quantified in five random fields from each slide at 400× magnification under a microscopy. Data shown on graph are means ± SD (*n* = 8 for all groups). ** *p* < 0.01 vs. WT. # *p* < 0.05 vs. its own control (PBS).

Consistent with clinical symptoms, histological analysis revealed epidermal hyperplasia, dermal edema, massive inflammatory infiltrates, and epidermal necrosis in DNFB-challenged mice at day 14 in WT and PAR1KO mice (Figure 2A). In PAR2KO mice, these features were much milder (Figure 2A). APC therapy reduced ear dorsum epidermal necrosis, dermal edema and inflammatory infiltrates in WT, and to a less extent in PAR1KO mice (Figure 2A). The thickness of ear epidermis and dermis and inflammatory infiltrates in PAR2KO mice were also marginally reduced in response to APC treatment (Figure 2A).

Mast cells (MCs) are principal effector cells in allergic diseases, including AD [27]. At day 14 after DNFB challenge, higher numbers of total/degranulated MCs were observed in all mice. However, when compared to WT mice, PAR1KO mice had significant lower numbers of both total and degranulated MCs, and PAR2KO mice also displayed lower number of degranulated MCs in the ear skin (Figure 2B). APC therapy inhibited MC infiltration and degranulation in WT, but not in PAR1KO or PAR2KO mice (Figure 2B,C), suggesting that the function of APC on MCs requires PAR1 or PAR2.

### 2.3. Effects of APC on Inflammatory Mediators in Mice with CHS

**Figure 3:**
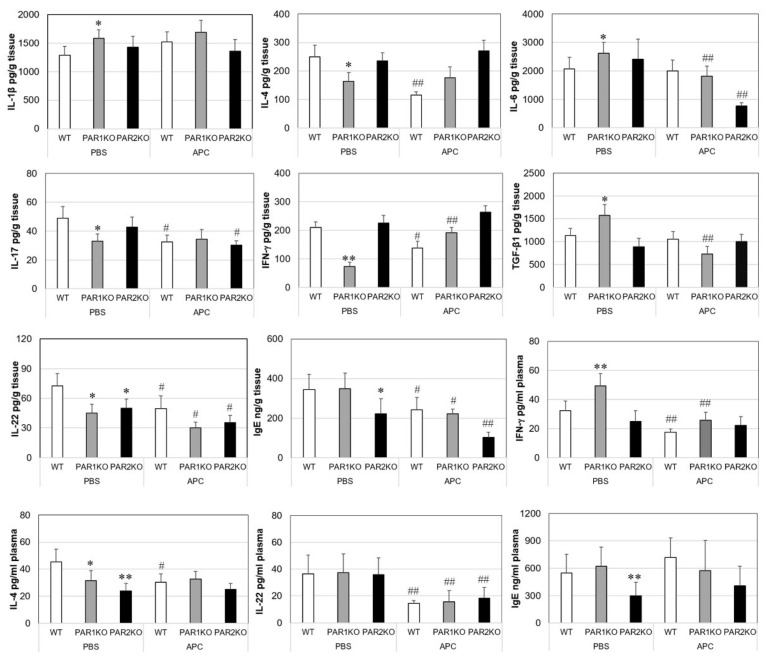
Cytokines and IgE in ear tissues or plasma from mice with CHS. Mice were euthanized at day 14 after DNFB challenge. Ear tissues were homogenized, cytokines and IgE in clear supernatants of tissue homogenates and plasma were measured by enzyme-linked immunosorbent assay. Data shown on graph are means ± SD (*n* = 8 for all groups). * *p* < 0.05, ** *p* < 0.01 vs. WT. # *p* < 0.05, ## *p* < 0.01 vs. its own control (PBS).

Inflammatory mediators were measured in both mouse ear skin tissue and plasma. In all mice without DNFB challenge, IL-1β, IL-4, IL-6, IL-17, IL-22, IFN-γ and TNF-α were not detectable and TGF-β1 was present at 150–170 pg/g ear tissue. No differences were found among different mouse strains. However, with the exception of TNF-α, all mediators were significantly increased in CHS ear tissue on day 14 (Figure 3). When compared to WT tissue, PAR1KO skin tissue contained higher levels of IL-1β, IL-6 and TGF-β1, and lower levels of IL-4, IL-17, IL-22 and IFN-γ, whereas most cytokines in PAR2KO ear tissue resembled those in WT tissue with only a lower IL-22 level (Figure 3). In the plasma, IFN-γ was higher and IL-4 lower in PAR1KO mice, and only a lower IL-4 level was observed in PAR2KO mice when compared to WT. APC therapy significantly reduced tissue levels of IFN-γ, IL-4, IL-17 and IL-22 in WT mice; IL-6, IL-22 and TGF-β1 in PAR1 KO mice; and IL-6, IL-17 and IL-22 in PAR2KO mice. Similarly, APC reduced plasma levels of IL-4, IL-22 and IFN-γ in WT, IFN-γ and IL-22 in PAR1KO mice, and IL-22 in PAR2KO mice. Plasma IL-17 was not detectable in any mouse.

IgE induced by various allergens can mediate the development of AD through activation of MCs and dendritic cells localized in the skin [27]. In this study, IgE was increased in both serum and ear skin tissue from mice with CHS. However, PAR2KO mice had 35% lower tissue and 45% lower plasma IgE when compared to WT and PAR1KO mice (Figure 3), which corresponds to its lower disease activity. APC therapy significantly reduced IgE levels in tissues, but not in plasma from all three mouse strains.

### 2.4. Effect of APC on Th1/Th2/Th17/Th22/Treg Phenotypes in Mouse Spleen Cells In Vitro 

**Figure 4:**
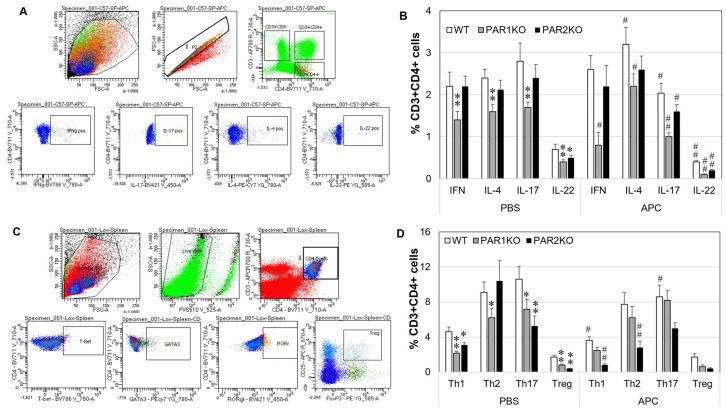
Intracellular Th cytokines and Th phenotypes in spleen cells from mice treated with APC in vitro. Spleen cells obtained from mice euthanized at day 14 after DNFB challenge were treated with APC (10 µg/mL) for 24 h. Cells were collected for detection of intracellular Th cytokines and Th phenotypes by flow cytometry. (**A**,**C**) Gating strategies for Th cytokines (**A**) and Th cells (**C**). (**B**,**D**) The percentages of IFN-γ, IL-4, IL-17 and IL-22 associated Th cells (**B**), and Th1, Th2, Th17 and Treg cells (**D**) in CD3 + CD4+ spleen lymphocytes. Data on graph are shown as mean ± SD (*n* = 3 independent experiments). * *p* < 0.05, ** *p* < 0.01 vs. WT. # *p* < 0.05, ## *p* < 0.01 vs. its own control (PBS).

APC and PAR1 or PAR2 deficiency have been shown to affect Th cell phenotypes in a mouse model of human rheumatoid arthritis [14,28]. In this study, the effect of APC and the deficiency of PAR1 or PAR2 on the specific intracellular Th cytokines and Th cell phenotypes were examined using spleen cells isolated from mice with CHS. The gating strategies were shown in Figure 4A,C. In control cells, compared to WT, percentages of IFN-γ (Th1), IL-4 (Th2), IL-17 (Th17) or IL-22 (Th22) associated CD3 + CD4+ T cells were lower in PAR1KO cells; in contrast, only a lower level of IL-22-associated CD3 + CD4+ T cells was observed in PAR2KO cells (Figure 4B). APC increased the percentages of IL-4, while decreased the percentages of IL-17 and IL-22 associated CD3 + CD4+ T cells in all three types of spleen cells. Additionally, APC decreased IFN-γ associated CD3 + CD4+ T cells in PAR1KO spleen cells. Th1/Th2/Th17 and Treg cells phenotypes identified by their nuclear factors in combination with cell surface markers were also investigated. CD3 + CD4+ T cells from PAR1KO spleen cells contained lower proportions of Th1/Th2/Th17/Treg cells, while PAR2KO spleen cells consisted of lower Th1/Th17/Treg cells, when compared to WT (Figure 4D). APC decreased Th1 and Th17 cells in WT cells, had no effect in PAR1KO cells, and decreased Th1 and Th2 cells in PAR2 KO cells (Figure 4D). These data indicate that APC may require PAR1 to exert its function on Th1/Th17/Treg cells.

### 2.5. The Expression of PAR1 and PAR2 in Skin Epidermis 

**Figure 5:**
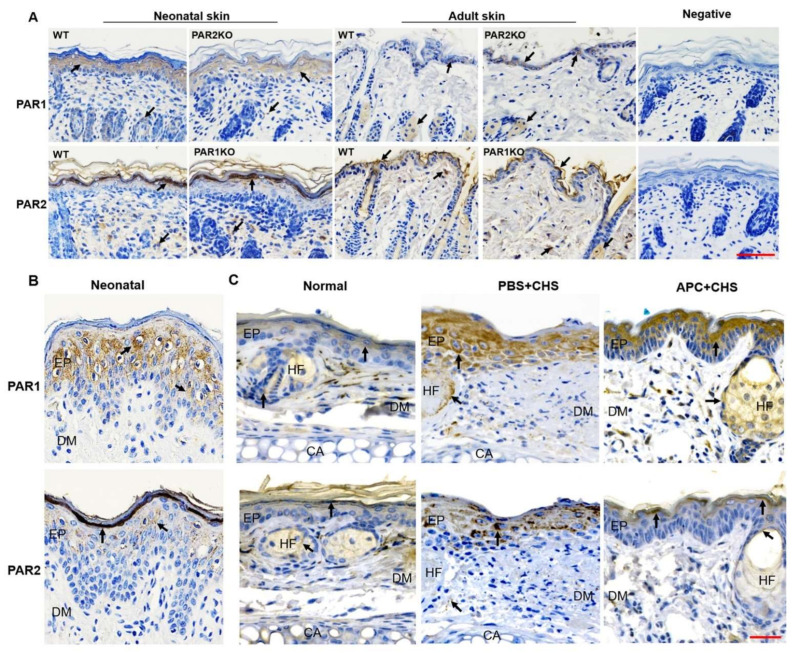
PAR1 and PAR2 expression in skin epidermis. PAR1 and PAR2 in the skin tissues were detected by immunostaining. (**A**) Representative images of PAR1 and PAR2 in skin tissues from newborn or adult WT, PAR1KO or PAR2KO mice. Scale bar: 100 µm. (**B**,**C**) Representative images of PAR1 and PAR2 in human neonatal foreskin (**B**), or in the ear skin from normal WT mice or WT mice with CHS treated with APC (2 mg/mL, APC + CHS) or PBS (PBS + CHS). Scale bar: 50 µm. CA: cartilage; EP: epidermis; DM: dermis; HF: hair follicle. Arrows indicate positive staining.

Skin barrier dysfunction is central to AD [29], and this barrier mostly depends on the epidermis. In newborn mouse skin (one to three days old when skin hair is yet to appear), PAR1 was present in the stratum granulosum and spinosum where viable keratinocytes are proliferating and differentiating, whereas PAR2 was present in the upper layers of the epidermis including the stratum corneum, lucidum and granulosum, where keratinocytes are terminally differentiated and cornified (Figure 5A). PAR1 and PAR2 in human neonatal foreskin epidermis showed similar expression patterns as shown in newborn mice (Figure 5B). In adult mouse skin (eight-week-old), although the epidermis was much thinner and epidermal layers were not distinct, PAR1 was present in keratinocytes with nuclei and PAR2 at the most upper layer of epidermis (Figure 5A). In addition, PAR2KO newborn mouse skin epidermis expressed less while adult skin expressed more PAR1 when compared to WT. There was no difference in PAR2 expression between PAR1KO and WT epidermis (Figure 5A). In the normal ear epidermis, the expression of PAR1 and PAR2 was relatively low. In response to DNFB challenge, this expression increased dramatically, and spread through the whole epidermis (Figure 5C). Interestingly, APC therapy not only suppressed PAR2 expression, but restored PAR1 and PAR2 normal expression patterns with PAR2 appearing in the upper layers of the epidermis and PAR1 retreating away from the basal layer (Figure 5C).

### 2.6. The Effect of APC on Mouse Keratinocytes In Vitro

**Figure 6:**
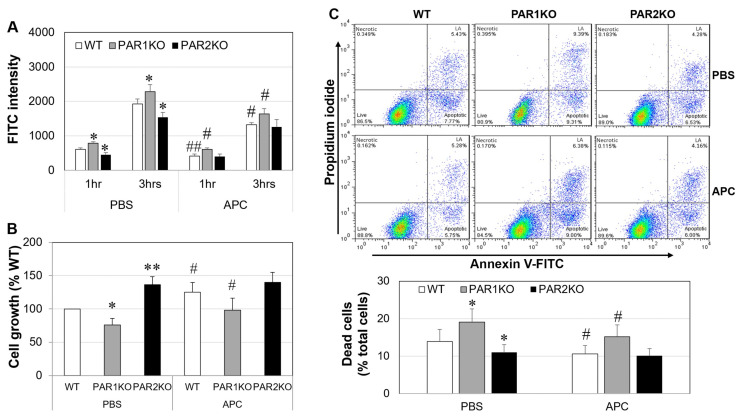
The effect of APC on barrier function, cell proliferation and apoptosis of mouse keratinocytes in vitro. Skin keratinocytes were isolated from newborn mice. (**A**) FITC intensity in the medium from lower wells of keratinocyte monolayers treated with APC (1 μg/mL) for 24 h, detected by fluorescence absorbance using a spectrometer. (**B**) The growth of keratinocyte treated with APC (1 µg/mL) for 72 h, detected by the MTT assay. (**C**) Percentage of dead cells (necrotic plus apoptotic cells) in keratinocytes treated with APC (1 µg/mL) for 24 h, detected by double staining using propidium iodide and Annexin V-FITC and a flow cytometer. Data on graph are shown as mean ± SD (*n* = 3/4 independent experiments). * *p* < 0.05, ** *p* < 0.01 vs. WT. # *p* < 0.05, ## *p* < 0.01 vs. its own control (PBS).

The barrier function of mouse keratinocytes was measured via the flux of FITC-dextran permeating from the apical to the basal side of keratinocyte monolayers. Under basal condition, compared to WT, FITC intensity was significantly higher in PAR1KO, and lower in PAR2KO cell monolayers at 1 and 3 h, respectively (Figure 6A). APC (1 µg/mL) reduced the FITC intensity of WT and PAR1KO, but not PAR2KO cell monolayers. In culture, PAR2KO keratinocytes grew significantly faster (~36%, *p* < 0.01), and PAR1KO cells much slower (~25%, *p* < 0.05), when compared to WT cells (Figure 6B). APC (1 µg/mL) increased WT and PAR1KO cell growth (~25%) but had no effect on PAR2KO cells (Figure 6B). When keratinocytes were cultured in chelex treated fetal bovine serum (chFBS)-reduced (1%) medium and detected by Annexin V and propidium iodide (PI) double staining and flow cytometry, PAR1KO resulted in a 37% increase (*p* < 0.05), while PAR2KO had a ~21% decrease (*p* < 0.05) in dead (apoptotic plus necrotic) cells compared to WT cells. APC reduced the number of WT and PAR1KO dead cells by ~20% (each *p* < 0.05) but did not affect PAR2KO cells (Figure 6C).

## 3. Discussion

This study demonstrates that elimination of PAR2 inhibited the development of NDFB-induced CHS in mice, while deletion of PAR1 had no significant effect on this disease. APC exerted a therapeutic benefit in both WT and PAR1KO mice but only had a marginal effect on CHS in PAR2KO mice. These results suggest that the therapeutic function of APC in NDFB-induced CHS is likely achieved mainly by suppressing PAR2. 

Similar functions of PAR1 and PAR2 have also been shown in experimental periodontitis, in which no difference is observed between WT and PAR1KO mice, whereas less alveolar bone resorption occurs in PAR2KO mice [30]. Interestingly, genetic elimination of PAR1 but not PAR2 attenuates disease severity in mouse models of human psoriasis [31]. These opposing effects of PAR1 and PAR2 on AD and psoriasis are probably due to the different underlying disease mechanisms of these two skin conditions. Although both diseases are characterized by epidermal hyperplasia and excessive inflammation, chronic AD is marked by the activation of Th1 cells alongside Th2 and Th22 cells, and IL-17 has a limited role; psoriasis however is a Th17-centric disease, with Th1 probably being a bystander [32]. In addition, PAR1 signaling on macrophages is required for oxazolone-induced CHS in mice [33]. The different roles of PAR1 in these two CHS models may be attributed to the different involvement of immune cells in disease pathogenesis. DNFB normally induces a Th1 cell-mediated response, whereas oxazolone results in a neutrophil dominated immune response [34]. Such contrast functions of PAR1 have also been reported in mouse gastral disease models. In C. rodentium-induced colitis, PAR1 promotes disease progression by stimulating Th17-related immunity [35], whereas, during H. pylori infection, PAR1 protects the host against severe gastritis partly via inhibition of neutrophil infiltration [36]. Furthermore, in mouse models of human arthritis, lack of PAR1 or PAR2 exacerbates collagen-induced arthritis but reduces the severity of antigen-induced arthritis [14]. These studies reveal complex disease specific roles of PAR1 and PAR2 in modulating the immune response. 

The mechanism behind these diverse functions of PAR1 and PAR2 at different inflammatory conditions are not clear but probably due to their biased signaling activated by different proteases [37,38]. For example, thrombin mediated PAR1 activation induces both endothelial and epithelial apoptosis and barrier disruption [38,39], while APC associated PAR1 cleavage promotes cell survival and barrier integrity [38,40]. Biased signaling is also found in PAR2. Trypsin cleavage of PAR2 leads to calcium mobilization, ERK1/2 and Rho activation, and β-arrestin1/2 recruitment [41]; cathepsin S cleavage of PAR2, however, prevents these actions [42]. In addition, the mechanism of co-receptor signaling also plays a key role in determining the specificity of PAR signaling. In endothelial cells, preoccupation of endothelial protein C receptor by protein C switches a barrier disruptive effect induced by PAR2 agonist to a barrier protective outcome [43]. Furthermore, the functions of PARs show cell/tissue specific mechanisms. Boucher et al. [44] demonstrate that myeloid-associated PAR1 promotes while intestinal epithelial associated PAR1 limits mucosal damage in C. rodentium-induced colitis in mice. The physiologic outcomes of PAR1/2 activation/inhibition therefore not only depend on specific agonist/ligand activation mode, but also cell type and the interaction with other receptors [37,38,40]. 

AD is featured by abnormal inflammation and skin barrier defects [45]. It progressively involves Th1, Th2, Th17 and Th22 pathways and prevails from nonlesional to acute and chronic lesional skin [46,47]. Thses cells exert their function via secretion of their signature cytokines such as IL-4, IL-17, INF-γ and IL-22. As expected, these cytokines were significantly elevated in DNFB-induced CHS skin in the chronic phase (at day 14) in WT mice. Although PAR1KO mice displayed similar disease severity as WT mice, their cytokine levels were lower in skin and plasma, and their spleen cells contained less Th1/Th2/Th17/Th22 cells (Figure 4). Fewer Th1/Th17 cells were also found in PAR2KO spleen cells. These results suggest that PAR1 and PAR2 can regulate Th cell activation and differentiation, although these cells and their cytokines may not be essential for the development of CHS in PAR1KO mice.

MCs act as both effector and regulatory cells in AD [48]. In CHS, WT mice displayed increased infiltration and degranulation of MCs, while total and/or degranulated MCs were lower in PAR1KO and PAR2KO mice. This data is in agreement with previous studies showing that thrombin, the major activator of PAR1, induced extensive infiltration and degranulation of MCs in mouse skin [49]; and inhibition of PAR2 activation reduced the degranulation of MCs in mouse models of AD [25]. However, the reduction in MC infiltration and activation, like that in Th cells, did not prevent CHS development in PAR1KO mice indicating that these cells are not vital for NDFB-induced CHS in these mice. 

Skin barrier function primarily depends on the epidermis, and keratinocytes are the dominant cells in this layer [50]. In normal epidermis, PAR1 is present in viable keratinocytes whereas PAR2 locates in the upper non-viable layers of epidermis in both human and mouse skin (Figure 5). In CHS, this expression pattern was disturbed with PAR1 and PAR2 being highly and more homogenously expressed by the whole epidermis. This expression pattern of PAR2 has also been found in the skin from patients with AD [16], and overexpression of PAR2 in epidermis alone can drive barrier dysfunction, inflammation, and pruritus in mouse models of human AD [19,51]. Our in vitro data further demonstrates the beneficial effects of PAR2 elimination on keratinocytes (Figure 6). In contrast, deletion of PAR1 disrupted keratinocyte growth/survival and barrier integrity (Figure 6A). This data is supported by the evidence that activation of PAR1 increases, whereas activation of PAR2 decreases proliferation and differentiation of keratinocytes [52]. These differential effects of PAR1 and PAR2 on the keratinocytes may contribute at least partly to the different disease outcomes in these KO mice.

Overall, our results indicate that in WT mice, excessive Th1/Th2/Th22 cells and MCs may play a dominant role in CHS development. In PAR1KO mice, the defective keratinocytes and inflammatory cytokines other than Th1/Th2/Th17/Th22, such as IL-1β, IL-6 and TGF-β1, may contribute to CHS development. In PAR2KO mice, however, the enhanced keratinocyte barrier function, reduced activation of Th cells and MCs, and the reduction in inflammatory cytokines may lead to mitigated CHS in these mice.

Another significant finding in this study is that APC displayed a therapeutical benefit for DNFB-induced CHS. APC is a natural anticoagulant with strong anti-inflammatory and barrier protective functions, and has proven effective in limiting disease progression in various inflammatory diseases or animal models of these conditions [8]. In allergic conditions, APC suppresses antigen-induced histamine release from rat peritoneal MCs [53], decreases Th1 and Th2 cytokines, IgE and hyperresponsiveness in a mouse model of asthma [9,54], and reduces neutrophil influx and degranulation in allergic asthma patients [10]. Similarly, in CHS, APC exerted its anti-inflammatory function via suppressing MC infiltration and degranulation, inhibiting Th1/Th2/Th22 cells and their associated cytokines. APC is known to promote Treg cells [11,28], in the current study, however, APC decreased these cells. Although Treg cells are considered anti-inflammatory, these cells are positively associated with AD severity and reduced significantly post-treatment in AD patients in remission but not in patients with active AD [55]. APC’s effect therefore may be associated with this nature of Treg cells in AD.

APC’s effect on keratinocytes likely plays an important part in the inhibition of CHS. In culture, APC stimulated mouse keratinocyte proliferation, protected these cells from apoptosis, and promoted their barrier integrity, similar to its effects on human keratinocytes [40,56]. In CHS, APC therapy not only suppressed PAR2 expression by epidermal keratinocytes, but also limited its distribution to the upper epidermal layer (Figure 5). Since mice with PAR2 overexpression in epidermal keratinocytes alone spontaneously develop AD-like symptoms [19,51,57], this function of APC may significantly contribute to its therapeutic effect on CHS. 

APC mediates its diverse pharmacologic benefits mainly via PAR1 [12]. Indeed, APC’s effect on MCs and Th1/Th17/Treg cells relied on PAR1 as APC’s inhibitory effect on these cells was lost in PAR1KO mice. In keratinocytes, although APC still showed a protective role in PAR1KO cells, it does not rule out the requirement of PAR1, as APC was less effective on these cells when compared to WT cells. The overall effects of APC on PAR1KO cells probably results from the combined consequences of PAR1 deficiency and the suppression of PAR2 by APC. This may partly explain why APC’s therapeutical effect on CHS in PAR1KO mice was less effective when compared to WT mice. These data also indicate that PAR1 is not the main receptor required for APC’s protective effect in CHS. Instead, this beneficial effect brought by APC maybe mainly achieved by suppressing PAR2. This is supported by the evidence that APC therapy reduced PAR2 expression and the severity of CHS in WT/PAR1KO mice, while it had no further protective effect in PAR2KO mice; APC’s inhibitory role on MCs and Th1/Th2 cytokines required PAR2; lack of PAR2 or the addition of APC showed almost identical protective effects in keratinocytes in vitro. 

PAR2 is also required for APC mediated T cells inhibition in the graft-vs.-host disease in mice via inhibition of downstream MAP kinase EERK1/2 and p38 activation [58]. In contrast, PAR2 mediates APC signaling in MC3T3 via activation of p38 kinase [59]. This suggests cell-specific downstream signaling pathways mediated by APC via PAR2. Interestingly, biased PAR2 antagonists that selectively block certain signaling pathways, attenuate MC degranulation and collagen-induced arthritis in rats [60], activate ERK1/2 in rat kidney epithelial cells [37], and suppresses inflammation and itch in mouse models of AD [61]. APC’s effects resemble those shown by biased PAR2 antagonists in these conditions [28,56], although exact signaling mechanisms mediated by PAR2 remain to be delineated.

In summary, this study demonstrated that elimination of PAR2, but not PAR1 mitigated DNFB-induced CHS in mice. Furthermore, APC showed therapeutic benefit in this disease by acting mainly through PAR2 suppression. Since PAR2 is a major contributor to AD, pharmacologic inhibition of PAR2 has long been expected to be of high therapeutic value, but its application remains a great change due to complex mechanisms of receptor activation and signaling. APC has been used to treat various inflammatory diseases with proven effectiveness [8]. This study suggests that APC likely exerts its function as a more specific biased PAR2 antagonist to mitigate mouse CHS. Thus APC may hold a great promise for clinical treatments for AD and other inflammatory skin disorders. 

## 4. Materials and Methods

### 4.1. Animals and CHS Induction

Six to eight week-old female PAR1KO, PAR2KO and matched WT mice (all with a C57 background) were bred and obtained from Kearns Facility as described previously [14]. CHS in these mice was induced via sensitization with 30 μL of 0.5% DNFB (Sigma-Aldrich, Merck KGaA, Darmstadt, Germany) on the flank skin on day zero and day one, 15 μL of 0.3% DNFB on the dorsum of both ears on day five to seven, and 15 μL of 0.1% DNFB on the ears on days eight–13 [26]. Mice received the same volume of the solvent (acetone and olive oil: 4:1, *v*/*v*) at the same time points were used as non-CHS negative controls.

### 4.2. Treatment

Mice were treated with recombinant human APC (2 mg/kg, Xigris, Eli Lilly Australia Pty Ltd. West Ryde NSW), PAR1 agonist (TFLLR-NH2) or its SC (FTLLR-NH2) (0.2, 2, 10, 40 and 80 mg/kg), or phosphate-buffered saline (PBS) at a volume of 200 µL/mouse via intraperitoneal injection daily 2 h prior to DNFB administration from day two to five as a preventative treatment, or from days eight to 12 as a therapeutic treatment. APC dose was optimized by our previous studies in mice [11,28].

### 4.3. Clinical Evaluation of CHS

Clinical severity of CHS was assessed by measuring ear thickness using a digital micrometer at day 0 and day three, then daily from day five to 14. At day 14, mice were euthanized, and ears, blood and spleens were harvested for further investigation.

Use of animals and all procedures were approved by Northern Sydney Health District Animal Ethics Committee.

### 4.4. Histological Examination

After euthanization, the flank skin (2 cm × 2 cm) from newborn (one to three day-old) or normal adult (eight-week-old) female mice, and ear tissue from mice with CHS were fixed in 10% PBS buffered formalin for 24 h and embedded in paraffin. Paraffin-embedded blocks were cut to a thickness of 4 μm and the tissue sections stained with H&E and toluidine blue for the histological examination and the quantification of MC infiltration and activation, respectively. MCs were identified by metachromasia in toluidine staining (due to the presence of highly sulfated proteoglycans) and evaluated in five random fields for each tissue section under a microscopy at the magnification of 400. All measurements were performed under blinded conditions by two or three independent researchers.

### 4.5. Th Cytokine and Th Cell Phenotype Detection

Spleen cells were treated with APC (10 µg/mL) for 24 h. 5 h prior to termination of the experiments, cells were stimulated with PMA (20 ng/mL)/Ionomycin (1 mM) in the presence of Monensin (5 µM). After stimulation, cells were collected and stained using a mouse antibody panel including CD3-AF700, CD4-BV711, IFN-γ-BV786, IL-4-PECY7, IL-17-BV421 and IL-22 (PE) (BD Biosciences, Franklin Lakes, NJ, USA) to detect Th cytokines in CD3+ and CD4+ lymphocytes. For specific Th cell phenotype identification, cells were treated with APC for 24 h and then stained with an antibody panel containing CD3-AF700, CD4-BV711, T-bet-BV786 (Th1), GATA3-PECY7 (Th2), Roγ-BV421 (Th17), FOX3-PE and CD25-APC (Treg) (BD Biosciences). Detection was performed using a BD LSR Fortessa flow cytometer (BD Biosciences).

### 4.6. Cytokines and IgE detection

Ear tissues were pre-cooled in liquid nitrogen, then smashed with Mikro-Dismembrator (Sartorius Group, GmbH, Goettingen, Germany) and collected into protein extraction reagent (Thermo Fisher Scientific, Waltham, MA, USA). After vortex, the homogenates were centrifuged at 12,000× *g* for 30 min at 4 °C and the clear supernatants collected. IL-1β, IL-4, IL-6, IL-17, IFN-γ, TGF-β1 and TNF-α (R&D Systems, Minneapolis, MN, USA), IL-22 and IgE (Biolegend, San Diego, CA, USA) in the clear supernatants or mouse plasma were measured by enzyme-linked immunosorbent assays according to the manufacturers’ instructions.

### 4.7. Keratinocyte Isolation and Apoptosis Detection

Mouse skin keratinocytes were isolated from newborn mice as described previously [61]. Cells were cultured in EMEM containing 0.05 mM CaCl_2_, 9% chFBS, penicilln 100 units/mL, streptomycin sulfate 100 U/mL, 10 ng/mL mouse epidermal growth factor (Thermo Fisher Scientific), and 50% filtered mouse fibroblast conditioned medium which was collected from culture of newborn mouse skin fibroblasts using DMEN medium supplemented with 10% chFBS.

### 4.8. Immunohistochemical Staining

Human foreskins and mouse skin tissue sections were de-paraffinized and subjected to immunostaining using rabbit anti-human or mouse PAR1 antibody, goat anti-human or mouse PAR2 antibody (Santa Cruz Biotech, Dallas, TX, USA), or anti-rabbit or anti-goat IgG (negative controls), followed by in the appropriate ready-to-use secondary antibody, and then the Liquid DAB+ Substrate Chromogen System (Agilent DAKO, Santa Clara, CA, USA). Finally tissue sections were counterstained and mounted, and photographed under a microscope.

Usage of human skin tissues was in accordance with Northern Sydney Health District Human Ethics Committee.

### 4.9. Cell Proliferation and Apoptosis

Cell growth was measured by a colorimetric 3-[4,5-dimethylthiazol-2-yl]-2,5-diphenyl tetrazolium bromide (MTT) assay, and cell death (necrosis plus apoptosis) was detected by Annexin V and PI double staining (Thermo Fisher Scientific) and flow cytometry. The data generated by flow cytometry was divided in four regions corresponding to: (1) necrotic cells (Necrotic) which are PI positive and Annexin negative; (2) late apoptotic cells (LA) which are PI and Annexin positive; (3) live cells (Live) which are negative to both PI/Annexin V; (4) apoptotic cells (Apoptotic) which are PI negative and Annexin V positive (Figure 5C).

### 4.10. Measurement of Flux of FITC-Dextran

Cells were cultured till confluence on 24-well culture inserts (0.4-μm pore size), then treated with APC (1 µ/mL) for 24 h. The flux of FITC-dextran was measured as described previously [40] which represents the permeability of the keratinocyte monolayers.

### 4.11. Statistical Analysis

Comparisons were performed using the two-tailed Student’s t test or one- or two-way analysis of variance (ANOVA) as appropriate. Two-way repeated measures ANOVA was used for data sets involving ≥3 groups. Student’s *t* test was used for data comparing two data sets only. Data are expressed as mean ± SD unless otherwise indicated, *p* < 0.05 was taken to represent statistical significance. All statistical calculation was performed using GraphPad Prism8.

## Data Availability

The data presented in this study is contained within the article.
